# Modulation of Catalytic Activity in Multi-Domain Protein Tyrosine Phosphatases

**DOI:** 10.1371/journal.pone.0024766

**Published:** 2011-09-13

**Authors:** Lalima L. Madan, S. Veeranna, Khader Shameer, Chilamakuri C. S. Reddy, R. Sowdhamini, B. Gopal

**Affiliations:** 1 Molecular Biophysics Unit, Indian Institute of Science, Bangalore, India; 2 National Centre for Biological Sciences (TIFR), Bangalore, India; University of South Florida College of Medicine, United States of America

## Abstract

Signaling mechanisms involving protein tyrosine phosphatases govern several cellular and developmental processes. These enzymes are regulated by several mechanisms which include variation in the catalytic turnover rate based on redox stimuli, subcellular localization or protein-protein interactions. In the case of Receptor Protein Tyrosine Phosphatases (RPTPs) containing two PTP domains, phosphatase activity is localized in their membrane-proximal (D1) domains, while the membrane-distal (D2) domain is believed to play a modulatory role. Here we report our analysis of the influence of the D2 domain on the catalytic activity and substrate specificity of the D1 domain using two *Drosophila melanogaster* RPTPs as a model system. Biochemical studies reveal contrasting roles for the D2 domain of *Drosophila* Leukocyte antigen Related (DLAR) and Protein Tyrosine Phosphatase on *Drosophila* chromosome band 99A (PTP99A). While D2 lowers the catalytic activity of the D1 domain in DLAR, the D2 domain of PTP99A leads to an increase in the catalytic activity of its D1 domain. Substrate specificity, on the other hand, is cumulative, whereby the individual specificities of the D1 and D2 domains contribute to the substrate specificity of these two-domain enzymes. Molecular dynamics simulations on structural models of DLAR and PTP99A reveal a conformational rationale for the experimental observations. These studies reveal that concerted structural changes mediate inter-domain communication resulting in either inhibitory or activating effects of the membrane distal PTP domain on the catalytic activity of the membrane proximal PTP domain.

## Introduction

The activity of Protein Tyrosine Phosphatases (PTPs) is critical for the regulation of signaling networks that govern cell growth, differentiation and communication. Changes or defects in the activities of either tyrosine phosphatases or kinases substantially perturb signaling pathways resulting in different diseased pathologies [1]. In *Drosophila melanogaster*, five Receptor Protein Tyrosine Phosphatases (RPTPs) control the growth of retinal axons and influence their ability to contact specific zones and prevent midline crossing of longitudinal axons [2]. DLAR, PTP99A, DPTP69D, DPTP52F and DPTP10D are selectively expressed on the Central Nervous System (CNS) axons and growth cones in the *Drosophila* embryo [3]. Genetic studies on these five RPTPs reveal intriguing relationships amongst these proteins ranging from partial redundancy, collaboration or competition depending on the temporal and/or cellular context [2], [4].

DLAR (Drosophila Leukocyte-Antigen-Related-like) and PTP99A (Protein Tyrosine Phosphatase on Chromosome band 99A7-8) play a key role in intersegmental nerve (ISN) branch-point decisions. While the guidance decision of the ISN axons to navigate past their first branch point requires concerted activity of DLAR and PTP99A, the entry of the Segmental Nerve b (SNb) into the Ventrolateral Muscle field depends on the tightly modulated antagonistic actions of the two RPTPs [3], [4], [5]. These observations could be rationalized by a model wherein the synergistic action of DLAR and PTP99A relies on common substrates resulting in the transduction of identical downstream signals. The antagonistic roles of these two RPTPs perhaps depend more on the spatial context whereby different substrates and thus different downstream signaling routes are activated. This model, however, does not account for the influence of the membrane-distal PTP domain on the activity and substrate specificity of these bi-domain PTPs. Here, we report experimental data and computational studies that suggest that interactions between the two PTP domains of these RPTPs play a significant role in the catalytic activity and substrate specificity of the proteins.

A prominent feature of a PTP domain is the conserved active site cysteine that serves as a nucleophile to attack the phosphate of the phosphotyrosine residue. Also, a conserved aspartate residue acts as a general acid to provide its proton to the leaving group, resulting in the formation of a cystienyl-phosphate enzyme intermediate . This aspartate residue then acts as a general base and along with two conserved glutamine residues activates a water molecule to dislodge this intermediate releasing the inorganic phosphate [6]. In the case of double domain RPTPs, the phosphatase activity is localized to the membrane proximal domain (D1) in most cases, while the membrane distal domain (D2) is inactive [7]. Biological relevance of this inactive D2 domain has been experimentally explored in the case of the human LAR protein where the D2 domain is crucial for the recognition of the Insulin Receptor [8]. Domain swapping experiments further revealed that the *in vivo* activity and substrate preferences could be altered for human LAR when its D2 domain was exchanged with that of CD45 [9]. In the case of RPTPα, where both D1 and D2 domains are active, the phosphatase activity of the D2 domain is crucial for RPTPα to elicit its biological response [10]. These apparently contradictory findings suggest that the role of the D2 domain could vary substantially. In this study, the substrate specificity of the tandem PTP domains of DLAR and PTP99A were examined using tyrosine phosphorylated peptides. In the case of DLAR, an analysis of PTP domain-peptide interactions suggests that the D2 domain binds to substrate peptides with a higher affinity than the D1 domain. In PTP99A, however, the D2 domain binds the peptides with a much lower affinity, when compared to its D1 domain. Fluorescence spectroscopy experiments using small molecule probes highlight the differences in the phosphotyrosine binding pockets of the two domains of DLAR and PTP99A. Molecular dynamics simulations using models of DLAR and PTP99A explain the lack of catalytic activity in the DLAR and PTP99A D2 domains, while providing a rationale for their substrate interaction. Importantly, critical differences in the inter-atomic interaction network rationalize the differences in the catalytic activities seen for the DLAR and PTP99A PTP domains. These studies thus suggest that the silent D2 domains may have evolved to provide a balance between peptide-binding and peptide-dephosphorylation in bi-domain PTPs.

## Materials and Methods

### Cloning, expression and purification of the RPTP Catalytic Domains

**Figure 1:**
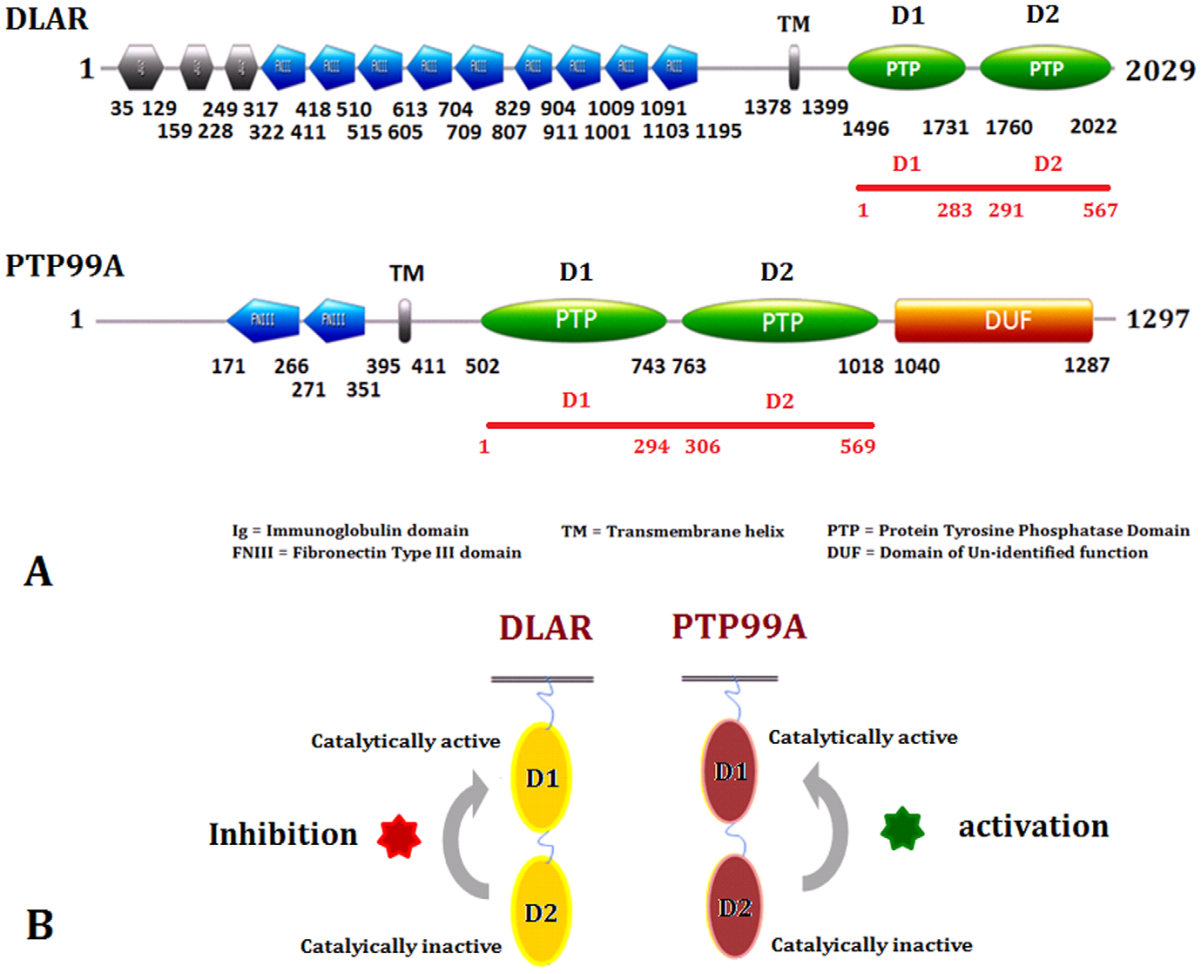
Domain architecture of DLAR and PTP99A RPTPs. A: Domain architecture of the DLAR and PTP99A proteins showing the extracellular domains and the intracellular PTP domain arrangement. The numbers in red refer to the amino acid numbering used for the models in the Molecular Dynamics Simulations. (Also listed in Table S5) B: Modulatory effects of the catalytically inactive D2 domain on the activity of the D1 domain of DLAR and PTP99A. While the D2 domain of DLAR decreases the activity of its D1 domain, the D2 domain of PTP99A causes an apparent increase in the activity of its D1 domain.

Cosmids containing the genes encoding RPTPs DLAR and PTP99A were a kind gift from Prof. Kai Zinn (Caltech). The PTP domains of PTP99A and DLAR were PCR amplified and ligated between the *Nhe*I and *Xho*I restriction sites of the bacterial expression vectors pET-15b and pET-22b. The active site mutants (with the nucleophilic cysteines in the PTP domains of DLAR and PTP99A mutated to serines) were obtained by using a single primer approach. An *XbaI* site was incorporated in the primers to aid screening of mutants. All clones were confirmed by sequencing (Macrogen Inc.). The details of the expression constructs are compiled in Table S1 and Figure 1a.

The plasmids containing the recombinant PTP domains were transformed into *E. coli* BL21(DE3) cells (Novagen, Inc.). Cells were grown to an optical density of 0.6 at 37°C in Luria Broth and induced with 0.1 mM IPTG. Following induction, the temperature for growth was lowered to 12°C and cells were grown for an additional 10–12 hrs before they were spun down and stored at −80°C. For purification of the recombinant PTP domains, cells were lysed by sonication in a buffer containing 50 mM Tris-HCl pH 8.0, 250 mM NaCl, 5% Glycerol and 1 mM β-mercaptoethanol (βME). The supernatant was incubated with His-Select Ni-NTA affinity resin (Sigma-Aldrich Inc.). After a wash with lysis buffer containing 20 mM Imidazole, the protein was eluted in 50 mM Tris-HCl pH 8.0, 250 mM NaCl, 1 mM βME and 200 mM Imidazole. The eluted protein was dialyzed overnight against 50 mM Tris-HCl pH 8.0, 50 mM NaCl and 1 mM βME. This was then loaded onto a Q-sepharose (GE Healthcare ) anion exchange column that had been pre-equilibrated with 20 mM Tris–HCl pH 8.0 and 1 mM βME. After extensive washing with Tris-HCl, the protein was eluted from the column by gradient of 0–1 M NaCl. Purity of the samples was assessed by SDS – PAGE. Concentrations of the proteins were accessed by their absorbance at 280 nm using their molar extinction coefficients.

### Identification of peptide substrates

Substrates of PTPs characterized thus far include the activation loop segments of various kinases and regulatory loops of other signaling proteins. Suitably designed peptide substrates mimic the physiologically relevant substrates of PTPs [11]. In the present study, peptide substrates were designed for DLAR for which more information was available on its interacting partners [2]. Five peptides viz., the Insulin Receptor peptide (TRDIpYETDYYR), Cuticle/PLC peptide (TAEPDpYGALYE), Nervous Fingers (VIGDpYVCRLCK), Myospheroid (CDDSpYFGNKC) and Abelson Peptide (RDDTpYTAHAG) were obtained from the PeptideMine server [12]. The five peptides were chosen based on their distinct charge distributions around the central phosphotyrosine residue. The phosphorylated as well as non-phosphorylated peptides were obtained from GL Bioscience, China. The peptides were >95% pure and were used after a single round of desalting using a Sephadex G25 column (GE Healthcare). The concentration of the peptide samples for biochemical studies was ascertained by UV absorption at 268 nm for the pY residue.

### Phosphatase assay using *para*-Nitrophenylphosphate (*p*NPP) and phosphotyrosine peptides

Phosphatase activity of the recombinant PTP domains was determined by using *para*-Nitrophenylphosphate (*p*NPP), a small molecule generic substrate, as well as phosphotyrosine containing peptides. The *p*NPP assay was performed as reported previously [13], with a modification in the buffer composition (25 mM Citrate, Glycine and HEPES (CGH), pH 6.5, 100 mM NaCl and 2 mM DTT). Phosphatase activity with the phosphotyrosine peptides was ascertained by the malachite green reaction as described for PTPs previously [14]. Briefly, the reaction mixture comprised of 0.01 µM protein incubated with increasing concentrations of different peptides in 25 mM citrate buffer pH 6.0, 100 mM NaCl and 2 mM DTT for 15 min at 25°C. The reaction was stopped by the addition of the Biomol Green reagent (Enzo Life Sciences) and the colour developed after 20 min was quantified at 650 nm. For all the assays, kinetic constants for the steady state catalysis were obtained by fitting the reaction curves to the Michaelis-Menten equation using the nonlinear regression module of Sigma plot software (Systat Software, Inc.).

### Ligand binding using *para*-Nitrocatechol sulfate (PNC) and phosphotyrosine peptides

5.0 µM of recombinant protein was titrated with increasing concentrations of PNC (Sigma-Aldrich) in 25 mM citrate buffer pH 6.5, 100 mM NaCl and 2 mM DTT. The closing of the WPD loop leads to the quenching of the intrinsic tryptophan fluorescence [15]. Fluorescence quenching was monitored at 350 nm (emission maximum) for recombinant PTP domains and the dissociation constants were calculated by fitting the data to a hyperbolic equation of ligand binding using the nonlinear regression modules of Sigma Plot software (Systat Software Inc.).

The binding of phosphotyrosine peptides to the PTP domains was studied by Surface Plasmon Resonance on a Biacore-2000 instrument (BIAcore, AB) [16]. All experiments were conducted at 25°C. DLAR PTP domains were immobilized onto carboxylated dextran CM5 chips (BIAcore AB) using the standard amine coupling procedure as recommended by the manufacturer. Binding and kinetic assays were performed in 10 mM HEPES, pH 8.0, 150 mM NaCl, 10 mM DTT and 3 mM EDTA at a flow rate of 5 µL/min. The peptides were used at concentrations ranging from 50–700 µM. Dissociation was initiated by replacing the analyte with buffer. The association and dissociation curves were monitored for 600 sec. Sensograms were analyzed with BIA-evaluation software version 2 (BIAcore AB). Sensograms for DLAR D1D2, DLAR D1HSSD2 and DLAR D1D2HSS were fitted to two-site binding equations. DLAR D1 and DLAR D2 sensograms were fitted using a single-site binding model.

### Homology modeling and *in silico* analysis of DLAR and PTP99A PTP domains

The sequences of the full length DLAR and PTP99A RPTPs were obtained from the Flybase database (FBgn0000464 and FBgn0004369 respectively). The different domains in the sequence were identified using the conserved domain database at the NCBI [17]. The sequences corresponding to the PTP domains of DLAR and PTP99A were used to search for homologues proteins using the Basic Local Alignment Search Tool (BLAST) server at the NCBI [18]. The MULTALIN server was used for performing multiple sequence alignments [19]. DLAR PTP domains were modeled on the structure of human LAR protein (PDB ID : 1LAR) and the PTP99A PTP domains were modeled on the crystal structure of RPTP sigma (PDB ID : 2FH7) using the MODELLER program for protein structure modeling [20]. The reliability of the model was checked by submitting the model to the WHAT-IF server [21]. The protein structures were visualized and superimposed using PyMOL software (DeLano Scientific LLC). The electrostatic potentials at the surface of the models were calculated by the Poisson-Boltzmann equation for supramolecular structures using the APBS software [22].

### Molecular Dynamics Simulation on models of DLAR and PTP99A

Homology models of various proteins have been used to study their conformational features by molecular dynamics [23]. The three-dimensional models of the DLAR and PTP99A PTP domains were examined using the GROMACS suite 4.0.7 (Groingen Machine for Chemical Simulations, [24]). In an effort to compare the PTP domains of DLAR and PTP99A directly with the previously reported PTPs, the residues in the homology models were re-numbered according to the PTP domain boundaries. This re-numbering is tabulated in Table S5. Briefly, the protein molecule was placed at the centre of a cubic box such that the edge of the box was at least 1.0 nm away from the molecule at the centre. The model was then solvated adding TIP4P waters to the system. The charge on the PTP was neutralized by adding appropriate numbers of either Na^+^ or Cl^−^ ions. The first derivative method of steepest descent in energy minimization was used to attain a potential energy minimum for the system before subjecting the same to MD simulations. Following *isothermal-isochoric* (NVT) and *isothermal-isobaric* (NPT) equilibration, the system was subjected to kinetic perturbations under the OPLS-AA/L all-atom force field of the GROMACS program at 300 K for 20 nanoseconds each. The Root Mean Square Deviation (RMSD) of the simulated structures from the initial structural model showed the system stabilizes by ca 7 nsec. All the structures were analyzed with snapshots taken between 10 nsec–20 nsec trajectory time. The Xmgrace software was used for numerical graphs and interpretation of data. Previously well studied closed and open forms of PTP1B (PDB IDs 1SUG and 2HNQ respectively) were used for the MD simulations primarily as a control model to evaluate the success of the simulations [25], [26], [27]. Cytoscape 2.3 was used to make the interaction map for the functionally important residues (FIRs) of the PTP domains [28].

## Results

### Characterization of the catalytic domains of DLAR and PTP99A

**Figure 2:**
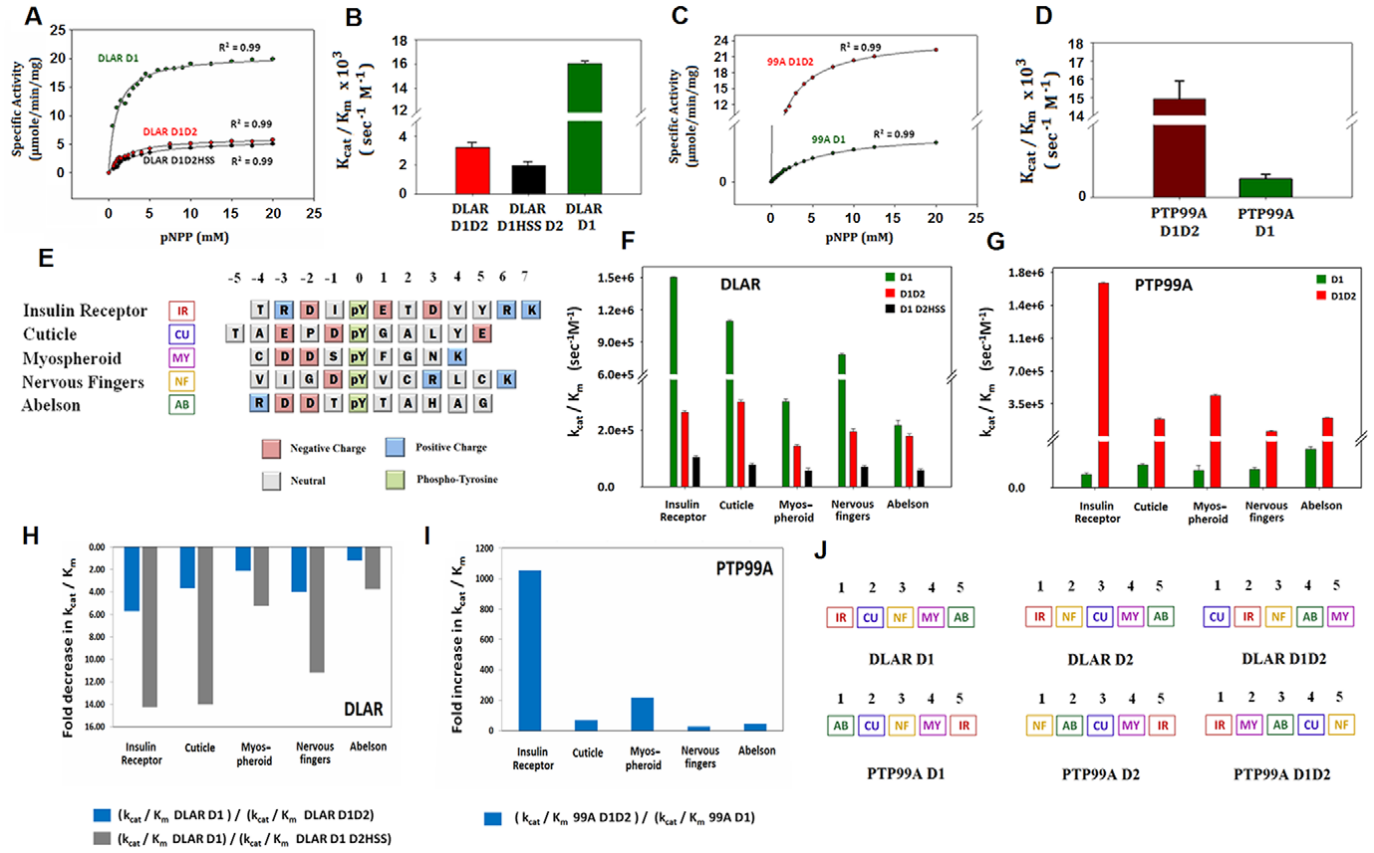
Phosphatase activity and substrate recognition features of the different DLAR and PTP99A protein constructs. A and B: Enzyme assay of the DLAR constructs using *para*-Nitrophenylphosphate (*p*NPP) as the substrate C and D: Enzyme assay of the PTP99A constructs using *p*NPP as the substrate; E : Peptide sequences used in the present study. Amino acids are highlighted by different colours to reflect their charge distribution; F and G : Kinetics of the dephosphorylation of various peptides by the different domains of DLAR and PTP99A; H and I : Fold change in the activity of the D1 domain due to the tethering of the D2 domain J : Ranking of the peptide substrates for the individual domains of DLAR and PTP99A.

The tandem PTP domains of PTP99A (PTP99A D1D2) and DLAR (DLAR D1D2) , the individual PTP domains (D1 and D2) as well as the active site mutants were expressed in *E. coli* and were purified using the protocols described earlier (Figure 1a, Figure S1a, b & c) [13]. The biochemical characterization and catalytic activity measurements were performed using *p*NPP as a substrate. Phosphatase activity measurements on tandem PTP domains of PTP99A and DLAR reveal that the PTPase activity in both PTP99A and DLAR was localized to the D1 domain, with no detectable activity in the D2 domain (Figure 1b, Figure 2a, b, c & d and Table S2). Mutation of the active site cystiene to serine in the D1 domain resulted in an inactive enzyme in both cases. Interestingly, while the activity of the DLAR D1 domain was much more (20.56 µmole/min/mg) than the D1D2 construct (6.19 µmole/min/mg), the activity of PTP99A D1 domain alone was much lower (0.22 µmole/min/mg) than that of the D1D2 protein (24.89 µmole/min/mg). Thus the D2 domain appears to have a regulatory role in both RPTPs- decreasing the catalytic activity in the case of DLAR while increasing the catalytic activity in the case of PTP99A (Figure 1b).

### Recognition and binding of substrate peptides to the PTP domains

The ability of the PTP domains of DLAR and PTP99A to dephosphorylate peptide substrates was used to evaluate the substrate recognition features of the two enzymes. DLAR and PTP99A were found to have distinct catalytic efficiencies for different peptide substrates (Figure 2e, f & g and Table S2). In the case of the DLAR D1D2 protein, the substrate preferences followed the order Cuticle peptide>Insulin receptor peptide>Nervous fingers peptide>Abelson peptide>Myospheroid peptide. PTP99A D1D2 showed a different ranking viz., Insulin Receptor peptide>Cuticle peptide>Myospheroid>Abelson peptide>Nervous fingers peptide. The observation that both the Cuticle peptide and the Insulin Receptor peptide were the most preferred substrates for both proteins suggests a similarity in DLAR and PTP99A substrate recognition. This observation is consistent with the finding that the synergistic action of these two proteins was required in some developmental contexts.

A comparison of the k_cat_/K_m_ of the D1D2 PTP constructs with that of the D1 domains of DLAR and PTP99A to obtain ratios of their catalytic efficiencies revealed inherent differences in the sequence specificity of their D2 domains (Figure 2 h, i & j). The peptide preference for the DLAR D1 domain was found to be Insulin receptor peptide>Cuticle peptide>Nervous fingers peptide>Myospheroid peptide>Abelson peptide. In the case of the DLAR D2 domain it was Insulin Receptor peptide>Nervous fingers peptide>Cuticle peptide>Myospheroid peptide>Abelson peptide. Similarly, the peptide preference of the D1 domain of PTP99A was seen to be Abelson peptide>Cuticle peptide>Nervous fingers peptide>Myospheroid peptide>Insulin receptor peptide while the D2 domain preferred Nervous fingers peptide>Abelson peptide>Cuticle peptide>Myospheroid peptide>Insulin receptor peptide. The difference between the most and least preferred substrate in the case of DLAR D1D2 was much less (∼16.0×10^4^ sec^−1^ M^−1^ between Cuticle and Myospheroid peptides) when compared to DLAR D1 alone (∼130.0×10^4^ sec^−1^ M^−1^ between Insulin Receptor and Abelson peptides). This suggests that the DLAR D2 domain plays a role in sequestering peptide substrates. The difference between the most and least preferred substrate in the case of PTP99A D1D2 was however much more (∼152.0×10^4^ sec^−1^ M^−1^ between Insulin Receptor and Nervous Fingers peptides) when compared to its D1 domain (∼30.0×10^2^ sec^−1^ M^−1^ between Abelson peptide and Insulin Receptor peptides) suggesting an activating role for its D2 domain. A more comprehensive study of this activation mechanism is being reported elsewhere.

**Figure 3:**
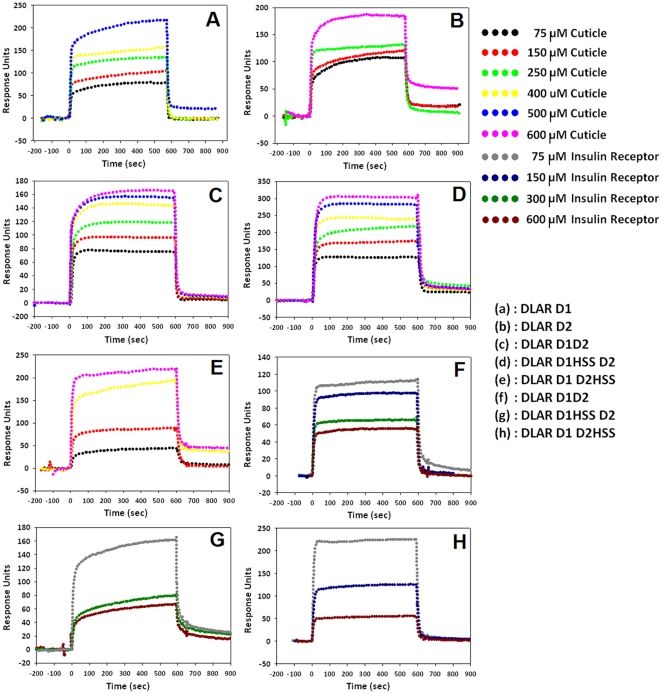
Representative Surface Plasmon Resonance (SPR) sensograms for the interaction between different DLAR constructs and phosphopeptide substrates. A: Interaction between DLAR D1D2 and the cuticle peptide, B: Interaction between DLAR D1HSS D2 and the cuticle peptide, C: interaction between DLAR D1D2HSS and the cuticle peptide, D: interaction between DLAR D1 and the cuticle peptide, E: interaction between DLAR D2 and the cuticle peptide F: Interaction between DLAR D1D2 and the Insulin Receptor peptide G: Interaction between DLAR D1HSSD2 and the Insulin Receptor peptide and H: Interaction between DLAR D1D2HSS and the Insulin Receptor peptide.

Surface Plasmon Resonance (SPR) experiments performed to evaluate the binding of peptide substrates to DLAR highlighted the role of the active site cystiene in peptide binding to the PTP domain. Another feature that was evident from the sensograms is that both Insulin Receptor peptide and the Cuticle peptide interactions for DLAR D1D2, DLAR D1HSS D2 (Cys to Ser mutation in the D1 domain (Figure S1 and Table S1) and DLAR D1D2HSS (Cys to Ser mutation in the D2 domain) could be fitted to a two independent sites binding model (Figure 3 and Table 1). For both peptides, it was seen that the binding to the second site (DLAR D2) was much stronger when compared to the first site (DLAR D1). A mutation in the active site cysteine of D2 to serine in DLAR D1D2HSS caused a thousand fold reduction in the association kinetics for both peptides. This reinforces the prominent role of the active site cystiene in binding the peptide substrates. A substantial difference in the K_D_ values for the first binding site for the DLAR D1HSS D2 construct (87.5 µM for the Cuticle peptide and 1.65 µM for the Insulin Receptor peptide) suggests that D1 is inherently more suited to bind the Insulin Receptor peptide. The D2 domain, in the absence of D1, does not bind the Insulin receptor peptide with the same affinity. We note that non-phosphorylated peptides did not interact with the immobilized proteins. This observation is consistent with the hypothesis that the phosphotyrosine residue is essential for PTP-substrate interaction.

**Table 1:** Parameters corresponding to the protein-peptide interactions.

Cuticle Peptide TAEPD(pY)GALYE						
	First Binding Site			Second Binding Site		
	k_a_ ( M^−1^ s^−1^)	k_d_ (s^−1^)×10^−4^	K_D_ (µM)	k_a_ (M^−1^ s^−1^)	k_d_ (s^−1^)×10^−4^	K_D_ (µM)
**DLAR D1D2**	32.6±1.57	102±4.80	312.8±0.12	1.65±0.23×10^4^	333±4.63	2.02±0.15
**DLAR D1HSS D2**	74.7±2.41	65.4±2.71	87.5±0.73	2.88±0.08×10^4^	540±2.17	1.87±0.03
**DLAR D1 D2HSS**	51.7±0.61	153±0.62	295.93±0.01	1.37±0.4	0.13±0.02	9.43±0.45
**DLAR D1**	18.6±1.46	17.6±4.37	94.6±0.33	–	–	–
**DLAR D2**	–	–	–	52.8±0.69	55.2±1.90	104.5±0.06

Insulin Receptor Peptide TRDI(pY)ETDYYRK						
	First Binding Site			Second Binding Site		
	k_a_ (M^−1^ s^−1^)	k_d_ (s^−1^)×10^−4^	K_D_ (µM)	k_a_ (M^−1^ s^−1^)	k_d_ (s^−1^)×10^−4^	K_D_ (µM)
**DLAR D1D2**	109.5±2.32	68.1±1.87	62.2±0.48	2.86±0.09×10^3^	0.61±0.07	2.13±0.46
**DLAR D1HSS D2**	72.6±1.20	1.20±0.21	1.65±0.19	74.5±1.60	1.40±0.09	1.87±0.08
**DLAR D1 D2HSS**	137±2.84	20.3±0.47	14.9±0.44	12.3±0.02	2.03±0.44	16.5±0.29

SPR sensograms were fitted to a single site binding model for single domain constructs and to independent two site binding model for the double domain constructs.

### Evaluation of the phosphotyrosine binding pocket

**Figure 4:**
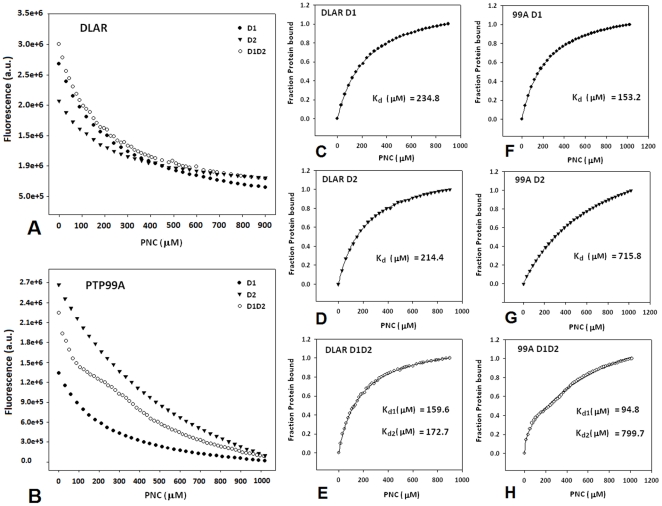
Evaluation of the phosphotyrosine binding pocket of the PTP domains of DLAR and PTP99A using *para*-Nitrocatecholsulphate (PNC). A and B: Fluorescence quenching upon PNC binding to constructs of DLAR and PTP99A ; C,D,F and G: binding of PNC to individual PTP domains ( D1 and D2) fitted to a single site site ligand binding model E and H: binding of PNC to tandem PTP domains (D1D2 constructs) fitted to the independent two site ligand binding model.

To evaluate the differences in the phosphotyrosine binding pockets of the two domains of DLAR and PTP99A, a small molecule probe *para*-Nitrocatecholsulfate (2-hydroxy-5-nitrophenyl sulfate; PNC) was used. PNC acts as a small molecule competitive inhibitor of the PTPs as it mimics the phosphotyrosine residue [15]. PNC binding was evaluated by monitoring the fluorescence quenching of the tryptophan residues in the WPD loop. These experiments show that the two PTP domains of DLAR bind PNC with comparable affinities (Figure 4a, c, d & e). PNC binding to the DLAR D1D2 construct fitted well to the independent ligand binding model for two non-interacting sites. In the case of PNC binding to PTP99A, PNC binding to the D1 domain of PTP99A alone was four times more favorable. The D2 domain of PTP99A bound PNC much more poorly when compared to the other PTP domains (Figure 4b, f, g & h).

### Structural rationale for the functional adaptation of PTP domains

**Figure 5:**
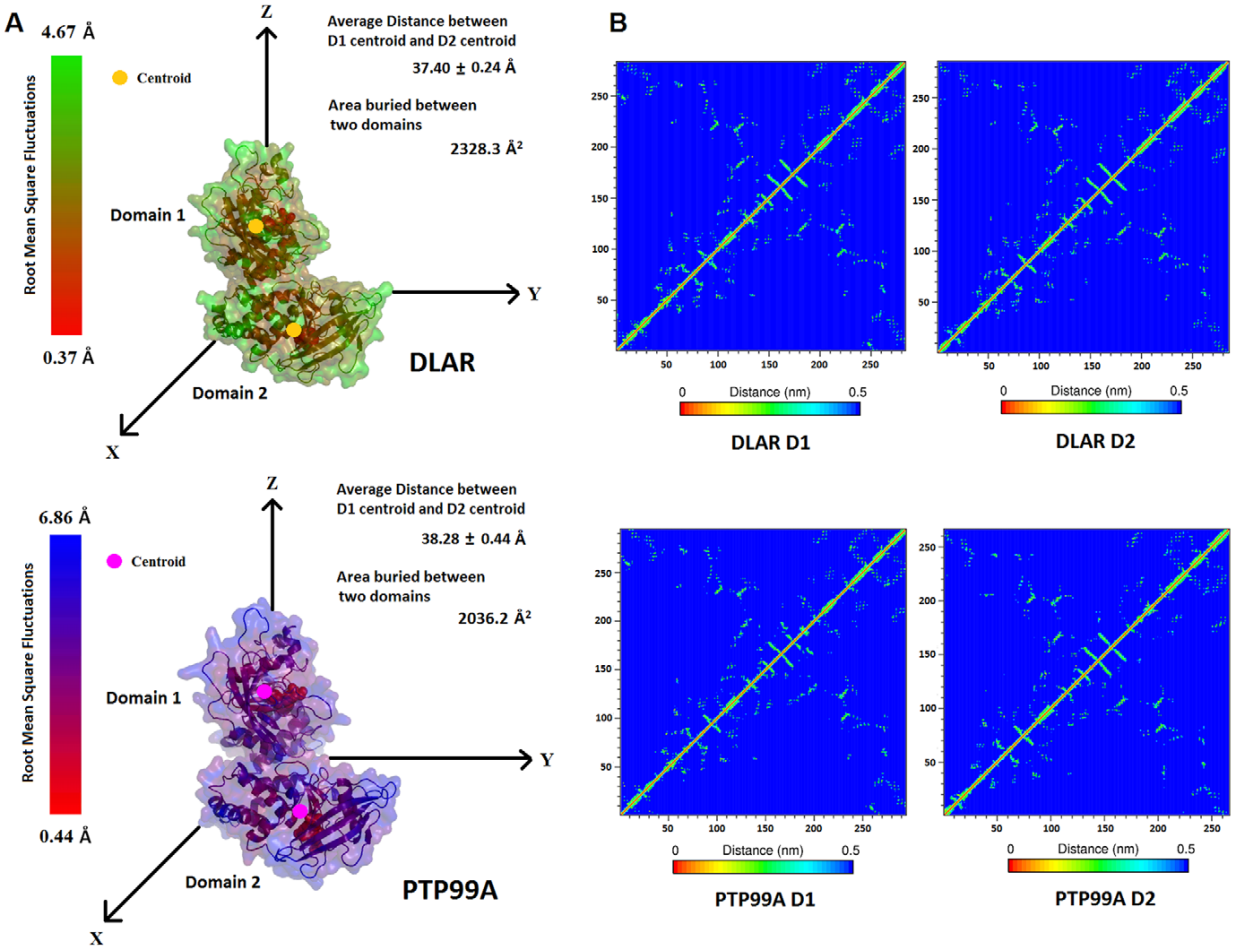
Inter-domain interactions and inter-atomic networks for the PTP domains obtained from molecular dynamics simulations. A: Root mean square fluctuations (RMSF) of each atom of the PTP domain in the simulation period mapped onto their 3D structures. Distance between the centroids of the D1 and D2 domains and the surface area buried between the two domains is also shown for each RPTP. B: Inter-atomic networks obtained for the individual PTP domains of DLAR and PTP99A.

Molecular dynamics simulations on PTP99A and DLAR provided vital insights into the interactions between the tandem PTP domains in double domain PTPs. The distance between the centroids of the two domains mapped over simulation time did not change much for both DLAR and PTP99A. The average distance between the centroids of the two domains of DLAR and PTP99A was 3.74±0.02 nm and 3.83±0.04 nm respectively (Figure 5a). The two PTP domains in each case are linked by a short polypeptide segment ( 12 aa in DLAR and 9 aa in PTP99A). This short linker between the two domains appeared quite rigid as seen by the minimal root mean square fluctuations for this segment during the MD simulations in both DLAR and PTP99A. This rigidity is likely to be conferred by the substantial buried surface area between the two domains (2328.3 Å^2^ and 2036 Å^2^ of buried surface area between the two domains of DLAR and PTP99A respectively).

The conformational segments corresponding to the ten conserved PTP motifs were analysed for structural changes during MD simulations by measuring the root mean square fluctuations for each C^α^ atom as well as for each atom per residue over the MD simulation time (Figure 5a, Tables S3 and S4). These fluctuations were also compared with those obtained for the PTP1B structures in the open and closed conformations. Among the ten motifs, the fluctuations were maximal for motif 8 containing the WPD loop. As this loop must close upon substrate binding, the flexibility of this loop is essential for the phosphatase activity of the PTP domain. Overall, more fluctuations were observed for the D2 domains of both DLAR and PTP99A. The phosphotyrosine binding motif (motif 1) showed substantial fluctuations which were more pronounced for the D2 domains as opposed to the D1 domains of both DLAR and PTP99A. Overall, the motifs 2, 3, 4, 5, 6 and 7 showed less fluctuations when compared to other motifs (motif 1, 8 , 9 and 10) for both domains of DLAR and PTP99A. Their lower kinetic fluctuations are perhaps expected, given that these motifs play a very important role in the folding and stability of the PTP domains [7].

### Inter-atomic networks reveal differences in the conformational dynamics of PTP domains

Spatially proximal residues (with inter-atomic distances less than 5 Å between residues averaged over the entire simulation time) were represented as N×N colour coded matrices (N = number of residues in the protein). All the six PTP domains examined showed a similar matrix which could be interpreted as a signature of the PTP fold. This inter-atomic interaction map has a shape like a butterfly where the body and wing comprise of interactions concentrated around the motifs 2, 3, 4, 5, 6 and 7 which form the core of the PTP domain (Figure 5b). The head of the butterfly pattern is made up of motif 1 whereas motifs 8, 9 and 10 form the tail. Interestingly, for all the six PTP domains analyzed, the body and the wings of the butterfly signature remain unperturbed. For the D1 domains of DLAR and PTP99A, the most striking differences were seen in the tail and head region. This feature perhaps best represents the differences in the peptide binding pockets of these PTP domains. A comparison of the matrices for the D1 and D2 domains of DLAR showed that these differences were localized to the head region (motif 1), few changes near the wing segments (interaction between motif 1 and motif 9/10) and fewer changes at the tail region (motifs 8 and motif 9). These differences in the inter-residue networks of the D2 domain rationalize the loss of activity in this PTP domain, although this domain is well folded and possesses all the ten conserved motifs that define a PTP domain.

**Figure 6:**
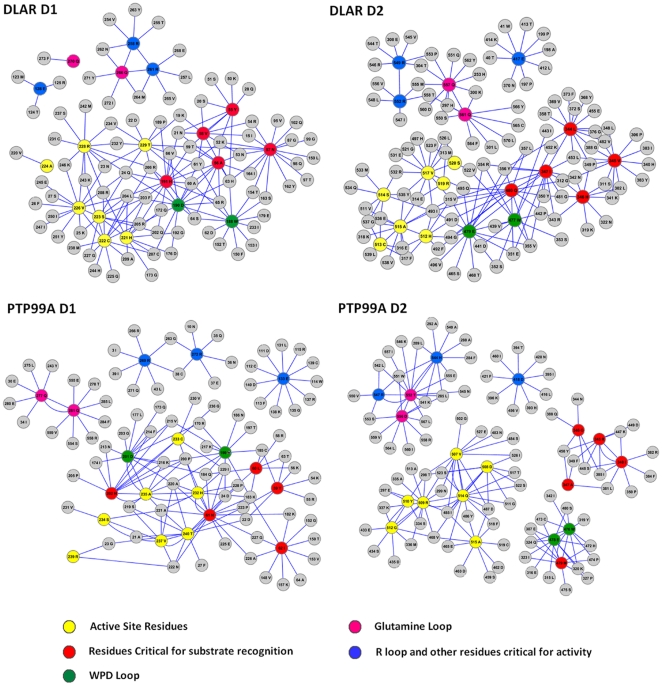
Interaction network of the twenty functionally important residues of the PTP domains of DLAR and PTP99A. Yellow: active site residues; red: residues critical for substrate recognition; green: residues of the WPD loop; pink: residues of the glutamine loop and blue: residues of the R loop and the conserved glutamate required for PTP activity.

The inter-atomic matrix provided a basis for an analysis of the interaction network between the 20 functionally important residues (FIRs) of the PTP domain (Figure 6). The FIRs can be divided into five groups: namely the active site residues, the WPD loop, the Glutamine loop, the R Loop and residues critical for substrate recognition and stabilization of the enzyme-substrate intermediate (Table S5). The interaction network clearly demonstrates that the active site residues, the WPD loop and the peptide recognition residues form a tight network amongst themselves in the DLAR and PTP99A D1 domains. This network is absent in the PTP99A D2 domain where the active site, the WPD and substrate recognition residues cluster separately. The glutamine loop, while important for the final step of addition of a water molecule in the dephosphorylation mechanism, clustered separately from the active site residues. The R loop and the conserved glutamate residue also clustered separately in the *Drosophila* PTP domains. While the active site of the DLAR D1 domain was tightly packed with a substantial number of non-covalent interactions with neighboring residues, the corresponding cluster in the D1 domain of PTP99A had fewer interactions. Also, in the D1 domain of DLAR, one of the glutamines of the Q loop was seen to interact with the R loop, while this interaction was absent in the D1 domain of PTP99A. Trp of the WPD loop of the D1 domain had more interacting partners and clustered differently when compared to the corresponding Trp residue of the D2 domain. These interaction networks thus reveal differences in the substrate binding and the active site hubs in the PTP domains of DLAR and PTP99A.

Molecular Dynamics simulations performed on the D1 domain alone of both DLAR and PTP99A revealed the effects of the presence of D2 on the interaction networks more explicitly (Figures S3 and S4). For a complete analysis of the MD data refer to http://caps.ncbs.res.in/download/dlar_ptp99a/). Importantly, both glutamines of the Q loop of the D1 domain of DLAR were seen to cluster with the R loop when the D1 domain was present in isolation. This interaction was uncoupled in the presence of the D2 domain of DLAR where one of the glutamines now clustered separately. This could perhaps account for the decrease in the activity of the D1 domain of DLAR in the presence of its D2 domain as it disrupts the glutamine network with the active site residues. Interestingly, in the case of PTP99A, the residues of the WPD loop formed a separate cluster from the active site when the D1 domain was present alone. This WPD loop cluster was seen to be merged with the active site residues in the presence of the D2 domain. It thus appears likely that the D2 domain of PTP99A enhances the activity of its D1 domain by strengthening the interaction networks between the active site residues and the WPD loop.

## Discussion

Differences in the functional roles of RPTPs have often been explained by sequence-structure variations as well as spatio-temporal effects in developmental processes. The role of extracellular domains of these RPTPs is clear from unambiguous genetic data - deletions in the Immunoglobulin–like domains of DLAR are lethal, while deletions in the Fibronectin type III repeats are not. The Fibronectin type III repeats are essential for *Drosophila* oogenesis suggesting that these domains are used in distinct signaling pathways and cell fate decisions in *Drosophila* development [29]. While the extracellular domains of these RPTPs are required for their proper localization in the nerve cell membrane, the signaling pathways at the growing axon cone are coordinated by the concerted activity of their cytosolic PTP domains.

The tandem PTP domains of double domain RPTPs form an interesting model system. In particular, the role of the catalytic D2 domain in the function of these proteins is unclear from genetic data. For example, the D1 domains of DLAR and DPTP69D have been examined for their ability to rescue the homozygous deletion mutations of these genes. In the case of DLAR, D1 was found to be redundant as D2 could itself partially rescue the DLAR −/− phenotype [29]. In the case of DPTP69D however, the active D1 domain was essential to rescue the DPTP69D −/− lethality [30]. These contradictory findings suggest a complex interplay between the PTP domains when attached in tandem.

A combination of biochemical studies using activity measurements, protein-substrate interactions and MD simulations were performed to understand the molecular basis of modulation of phosphatase activity in the two tandem PTP domains of DLAR and PTP99A. These studies reveal that the entire phosphatase activity in the two proteins is localized to their D1 domains. The presence of the D2 domains, however, leads to a change in their catalytic activity. Phosphatase activity, monitored using both *p*NPP and the phosphotyrosine peptide substrates, reveal that the D2 domain of DLAR has an inhibitory effect on its D1 domain while the D2 domain of PTP99A enhances the activity of its D1 domain. Substrate recognition features were also substantially influenced by the presence of the D2 domain in both cases. In the DLAR D1D2 construct, when the most preferred substrate of the D1 domain (the Insulin Receptor peptide) is sequestered by the D2 domain, the Cuticle peptide is preferentially de-phosphorylated. This perhaps explains the observation that D2 deletion constructs are significantly impaired in phenotypic rescue of the embryos [29]. The deletion of the D2 domain would impart the D1 domain of DLAR with much higher activity, but would alter its substrate recognition pattern leading to its inability to regulate signaling pathways. The biochemical data also reveals that the substrate recognition by the DLAR D1D2HSS construct is similar to the wild type DLAR D1D2 protein. This suggests that while the active site cysteine of the D2 domain is important for peptide binding, it does not dictate the target sequence recognition of the PTP domain. This observation is consistent with the finding that neuronal phenotypes of DLAR knock-outs could be rescued by the C1929S transgene of DLAR with comparable efficiency to that of the wild type DLAR in *Drosophila* embryos [29].

The D2 domain of PTP99A, while structurally conserved, has critical mutations in motifs 9 and 10 suggesting a loss of catalytic activity (Table S3). The active site Cys of this PTP domain is substituted by an Asp, which has been previously shown to be capable of substrate binding, but is deficient in catalysis [31]. A point mutation of this asp (Asp 958) to Cys alone could not activate the D2 domain of PTP99A suggesting that the presence of other motifs is crucial for catalytic activity in this class of proteins (Figure S5a, b & c). Interestingly, PTP99A is the only type III RPTP with a membrane distal D2 domain [7]. Electrostatic potentials at the surface of the PTP99A D2 domain highlight the negative charges, which are quite uncommon in the phosphotyrosine binding pocket of the PTP domains (Figure S2). On the other hand, the positive charges at the pY binding sites of the D1 and D2 domains are consistent with the competitive binding of substrates by the two domains of DLAR.

MD simulations of the PTP domain models were used to understand the conformational basis of the interaction between the two PTP domains of DLAR and PTP99A. As the linker connecting the two PTP domains is crucial for maintaining the substrate specificity of the LAR and LCA RPTPs [32], it was speculated that movements in the linker, could, in principle, play a role in communication between the two PTP domains. The positioning of the linker at the backside of the D1 domains is an evolutionary hotspot harboring the allosteric site for modulation of activity in single domain PTPs [33]. In the present studies, the minimal root mean square fluctuations in the linker region over simulation time suggests that the linker between the two domains is quite rigid. It thus appears likely that residues in the linker may not be solely responsible for domain-domain interactions.

To evaluate the role of other conserved protein segments in inter-domain interactions, the inter-atomic network of the PTP domains were examined for each residue (within 5 Å) for each PTP domain. While the butterfly pattern of the PTP fold was observed in all the four PTP domains, alterations in the networks of functionally important residues (FIRs) could rationalize the differences in the biochemical properties of the PTP domains. We speculate that the smaller clusters in the D1 domain of PTP99A compared to that of DLAR could be correlated with the low intrinsic activity of the PTP99A protein. Differences in the network between the active site Arg, the general acid Asp, the Trp at the hinge and the peptide recognition residues between the D1 domains of DLAR and PTP99A reflect the differences in their substrate recognition features. Substitution of two critical amino acids, leading to the loss of activity in the D2 domains of the LAR family (Motif 1 Tyr and the Motif 8 Glu) [34] is reflected in the alterations in their inter-atomic networks . While the D2 domain of PTP99A also shows the sequence signatures within the butterfly pattern of the PTP fold, the disjoint hubs of residues implicated in substrate binding and catalysis reveals smaller differences between this PTP domain and the others. This finding is consistent with the observation that the interaction networks based on the MD simulations of the D1 domain alone are different from that of the D1D2 proteins. The D2 domain of DLAR is quite similar to its D1 domain in sequence, a feature that is also reflected in their inter-atomic networks. On the other hand the D2 domain of PTP99A is not as similar to its D1 domain or the other PTP domains in sequence (Table S3). A different interaction network seen in this case suggests that this domain could have evolved as a modulatory domain to influence the activity of its catalytically active D1 domain.

A comparison of PTP sequences to understand the evolution of PTP domains suggests that the inactive D2 domains evolved from a common ancestor. The ancestor then appears to have delineated to form two subsets: one subset which accumulated mutations around the active site, and the other which accumulated mutations at its backside [35]. The studies presented here provide an example of each of these two lineages. While the D2 domain of PTP99A could be a prototype of the former, the D2 domain of DLAR falls in the latter category. The D2 domain of PTP99A has accumulated mutations around the active site, thereby losing phosphatase activity. The D2 domain of DLAR, on the other hand, accumulated mutations at the backside of the active site, in particular at motif 1 and motif 8, which allows the domain to bind substrate peptides but hinders phosphatase activity. Put together, these studies provide a model to understand the role of the tandem PTP domains in bi-domain PTPs.

## Supporting Information

Figure S1 **Purification profile of the recombinant PTP proteins used in the present study.** A: Schematic to show the different mutants used in the present study. B and C: Purified constructs of the catalytic domains of DLAR. Lane 1: DLAR D1D2, Lane 2: DLAR D1HSS D2, Lane 3: DLAR D1 D2HSS Lane 4: DLAR D1HSS D2HSS, Lane 5: DLAR D2, Lane 6: DLAR D2HSS, Lane 7: DLAR D1, Lane 8: DLAR D1 HSS, Lane 9: PTP99A D2, Lane 10: PTP99A D1, Lane 11: PTP99A D1D2, Lane 12: PTP99A D1 HSS, Lane 13: PTP99A D2, Lane 14: PTP99A D1HSS D2, M: Molecular weight marker.
(TIF)

Figure S2 **Surface electrostatic potential distribution in DLAR and PTP99A.** The electrostatic potential at the surface of DLAR and PTP99A as estimated by the APBS tool (Pymol software). The phospho-tyrosine binding pocket of each domain of DLAR and PTP99A is highlighted. Active site residues in the binding site are represented as sticks.
(TIF)

Figure S3 **Interaction networks for the 20 Functionally Important Residues (FIR) of the D1 domain of DLAR.** The interaction network for the FIRs were computed over 2 ns time scales for the D1 domain of DLAR in the presence and absence of its cognate D2 domain.
(TIF)

Figure S4 **Interaction networks for the 20 Functionally Important Residues (FIR) of the D1 domain of PTP99A.** The interaction network for the FIRs were computed over 2 ns time scales for the D1 domain of PTP99A in the presence and absence of its cognate D2 domain
(TIF)

Figure S5 **Characterization of the D958C mutant of the D2 domain of PTP99A.** A: Size exclusion profile of wild-type and D958C mutant of the D2 domain of PTP99A. B: Circular Dichroism (CD) spectra of the wild type and D958C mutant. The D958C mutation does not alter the secondary structure of the D2 domain. C: *para*-Nitrophenyl Phosphate assay for the phosphatase activity for different constructs of PTP99A. The D958C point mutant is catalytically inactive.
(TIF)

Table S1 **List of primers used for the cloning of the recombinant PTP domains of DLAR and PTP99A**
(DOC)

Table S2 **Kinetic parameters obtained for the dephosphorylation of various substrates by the active constructs of DLAR and PTP99A.**
(DOC)

Table S3 **Sequence motifs defining the PTP domain of DLAR and PTP99A.**
(DOC)

Table S4 **RMSF for the ten sequence motifs defining the PTP domains of DLAR and PTP99A.**
(DOC)

Table S5 **Residue numbers for the functionally important residues (FIR) as they occur in the sequence of DLAR and PTP99A, and as they are seen in the homology models of the RPTPs used in the Molecular Dynamics Simulations.**
(DOC)
