# Supplementary figures and images for: Modulation of Catalytic Activity in Multi-Domain Protein Tyrosine Phosphatases
Source: PLoS One. 2011 Sep 13;6(9):e24766. doi: 10.1371/journal.pone.0024766 (PMC3172300; doi:10.1371/journal.pone.0024766)

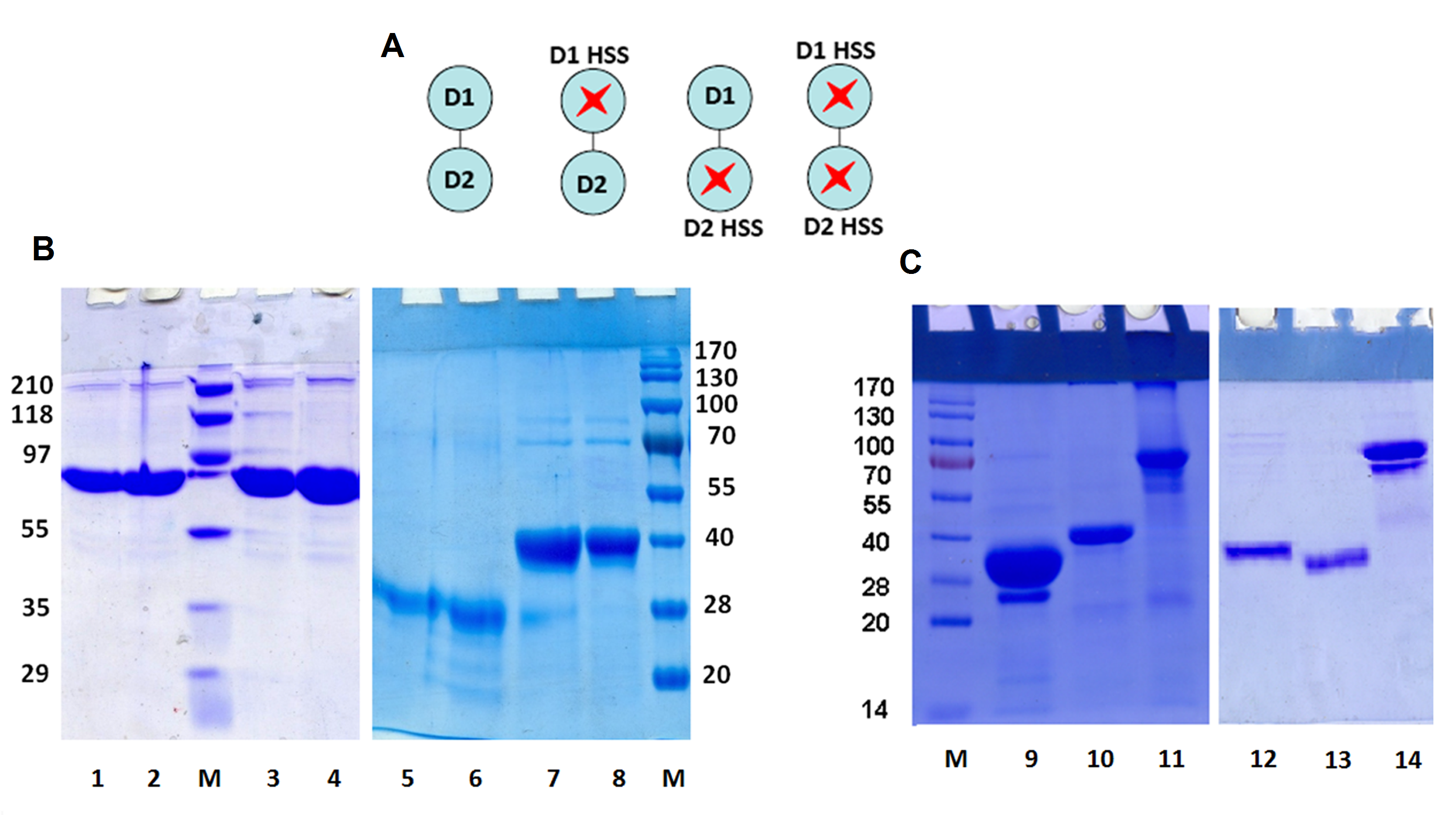

Supplement: Figure S1 — Purification profile of the recombinant PTP proteins used in the present study. A: Schematic to show the different mutants used in the present study. B and C: Purified constructs of the catalytic domains of DLAR. Lane 1: DLAR D1D2, Lane 2: DLAR D1HSS D2, Lane 3: DLAR D1 D2HSS Lane 4: DLAR D1HSS D2HSS, Lane 5: DLAR D2, Lane 6: DLAR D2HSS, Lane 7: DLAR D1, Lane 8: DLAR D1 HSS, Lane 9: PTP99A D2, Lane 10: PTP99A D1, Lane 11: PTP99A D1D2, Lane 12: PTP99A D1 HSS, Lane 13: PTP99A D2, Lane 14: PTP99A D1HSS D2, M: Molecular weight marker. (TIF) [file pone.0024766.s001.tif]

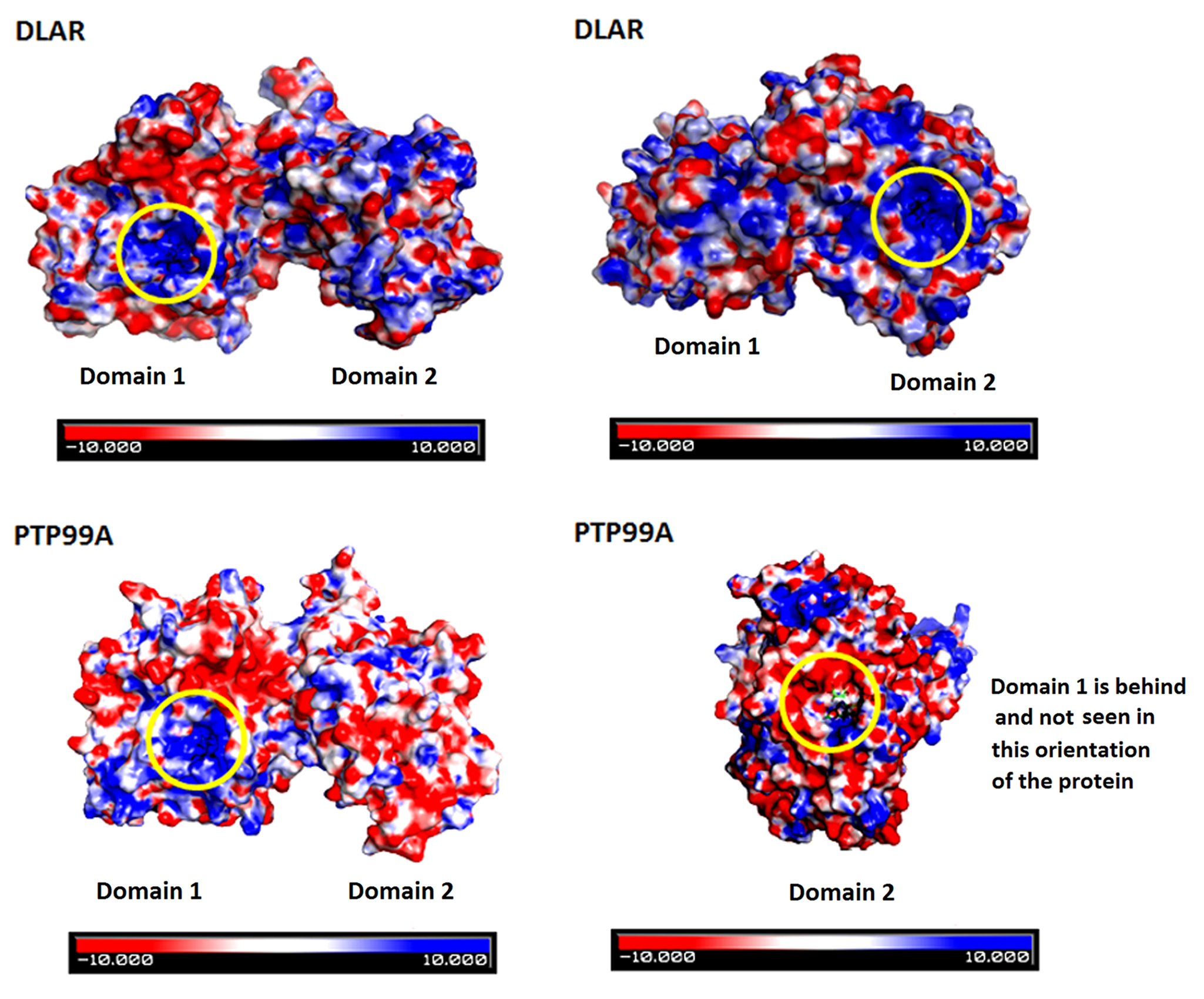

Supplement: Figure S2 — Surface electrostatic potential distribution in DLAR and PTP99A. The electrostatic potential at the surface of DLAR and PTP99A as estimated by the APBS tool (Pymol software). The phospho-tyrosine binding pocket of each domain of DLAR and PTP99A is highlighted. Active site residues in the binding site are represented as sticks. (TIF) [file pone.0024766.s002.tif]

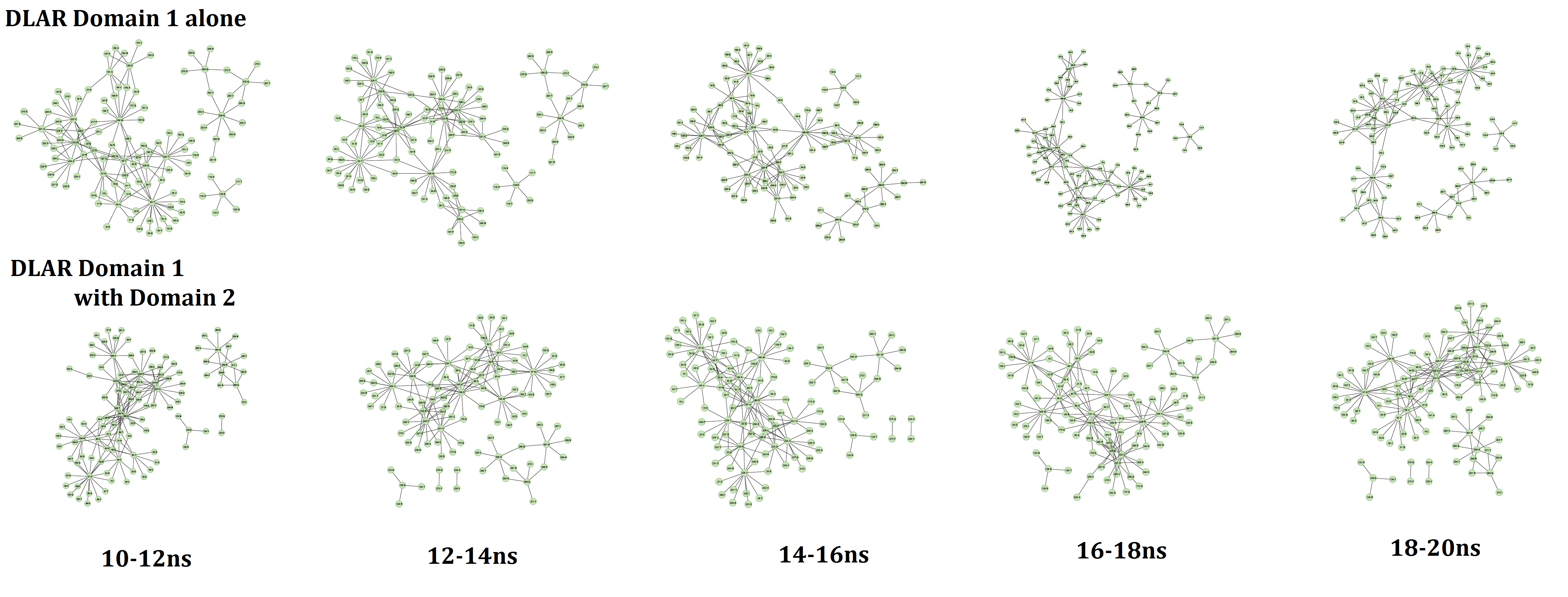

Supplement: Figure S3 — Interaction networks for the 20 Functionally Important Residues (FIR) of the D1 domain of DLAR. The interaction network for the FIRs were computed over 2 ns time scales for the D1 domain of DLAR in the presence and absence of its cognate D2 domain. (TIF) [file pone.0024766.s003.tif]

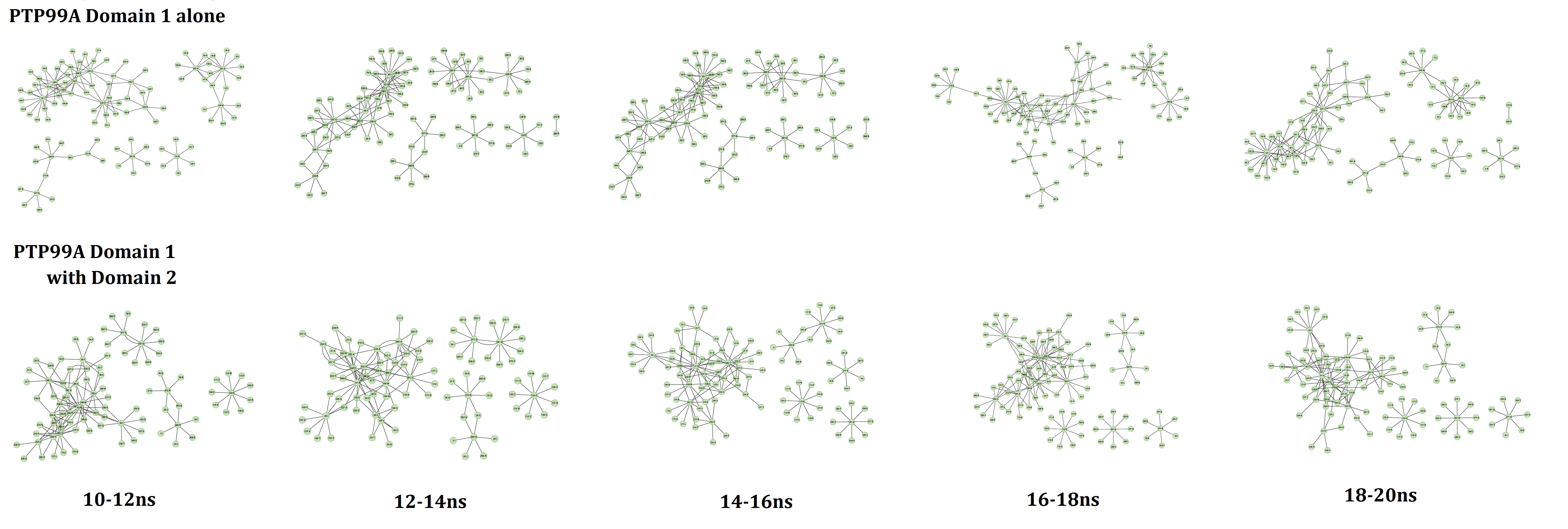

Supplement: Figure S4 — Interaction networks for the 20 Functionally Important Residues (FIR) of the D1 domain of PTP99A. The interaction network for the FIRs were computed over 2 ns time scales for the D1 domain of PTP99A in the presence and absence of its cognate D2 domain (TIF) [file pone.0024766.s004.tif]

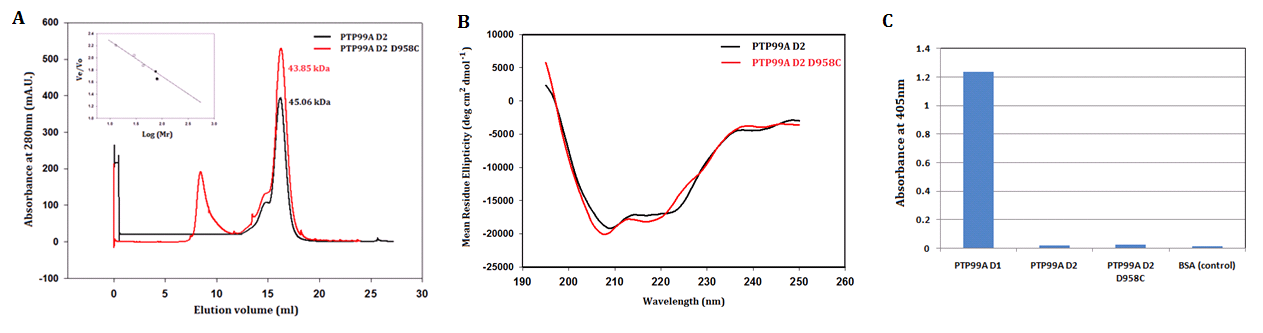

Supplement: Figure S5 — Characterization of the D958C mutant of the D2 domain of PTP99A. A: Size exclusion profile of wild-type and D958C mutant of the D2 domain of PTP99A. B: Circular Dichroism (CD) spectra of the wild type and D958C mutant. The D958C mutation does not alter the secondary structure of the D2 domain. C: para-Nitrophenyl Phosphate assay for the phosphatase activity for different constructs of PTP99A. The D958C point mutant is catalytically inactive. (TIF) [file pone.0024766.s005.tif]
