# Supplementary material for: Modulation of Catalytic Activity in Multi-Domain Protein Tyrosine Phosphatases
Source: PLoS One. 2011 Sep 13;6(9):e24766. doi: 10.1371/journal.pone.0024766 (PMC3172300; doi:10.1371/journal.pone.0024766)
Supplement: Table S1 — List of primers used for the cloning of the recombinant PTP domains of DLAR and PTP99A (DOC) [file pone.0024766.s006.doc]

**Table S1 :** **List of primers used for the cloning of the recombinant PTP domains of DLAR and PTP99A**

| **Primer Information for the cloning of the recombinant PTP domains of DLAR and PTP99A** | | | | |
| --- | --- | --- | --- | --- |
| Protein name as used in the manuscript | Protein construct | Primer Sequence  5’ ------ 3’ | | |
| DLAR D1 D2 | DLAR 1448-2029 | Forward primer | | TATA**GCTAGC**ATGATCTCCCATCCGC |
|  |  | Reverse Primer | | AATA**CTCGAG**GTTTGTATAATTGTCGAATGAGCCCAA |
| DLAR D1 | DLAR 1448-1746 | Forward primer | | AATA**GCTAGC**GTTAAGCGTCGTCGCCAGCCGTG |
|  |  | Reverse Primer | | AATA**CTCGAG**GTGGGTGTGTAGATTGCGAGC |
| DLAR D2 | DLAR 1728-2029 | Forward primer | | AATAA**GCTAGCG**CTCGCAATCTACACACCC |
|  |  | Reverse Primer | | AATA**CTCGAG**GTTTGTATAATTGTCGAATGAGCCCAA |
| PTP99A D1 D2 | PTP99A 450-1050 | Forward primer | | TATA**GCTAGC**GACGAGATCCGAGCGGCT |
|  |  | Reverse Primer | | TATA**CTCGAG**ACCATTGCTGCATATT |
| PTP99A D1 | PTP99A 450-756 | Forward primer | | TATA**GCTAGC**GACGAGATCCGAGCGGCT |
|  |  | Reverse Primer | | ATTA**CTCGAG**CTCCTCCACCTGCTCGGCCA |
| PTP99A D2 | PTP99A 740-1050 | Forward primer | | ATTA**GCTAGC**GAGGCCATCGCCTCGGGGGA |
|  |  | Reverse Primer | | TATA**CTCGAG**ACCATTGCTGCATATT |
| **Primers used for Mutagenesis** | | | | |
| Protein name as used in the manuscript | Protein construct | Primer Sequence  5’ ------ 3’ | | |
| DLAR D1 HSS | DLAR C 1670 S | Cycle I | GGACCCGTGATTGTTC**TCTAGA**CTGCGGGAGTTGGTCGCAC | |
|  |  | Cycle II | GGACCCGTGATTGTTCACTCCTCTGCGGGAGTTGGTCGCAC | |
| DLAR D2 HSS | DLAR C 1961 S | Cycle I | GGACCCATTACCGTGC**TCTAGA**CGGCGGGCGTGGGACGTT | |
|  |  | Cycle II | GGACCCATTACCGTGCACTCTTCGGCGGGCGTGGGACGTT | |
| PTP99A D1 HSS | PTP99A C 682 S | Cycle I | GGTCCCATAGTCGTGC**TCTAGA**GCGCTGGCGTCGGTCGC | |
|  |  | Cycle II | GGTCCCATAGTCGTGCACTCCAGCGCTGGCGTCGGTCGC | |

List of primers used for the cloning of the recombinant PTP domains of DLAR and PTP99A into *E.coli* expression vectors. Also listed are the primers used for the site directed mutagenesis to generate active site mutants of DLAR and PTP99A. Restriction sites are highlighted in bold :GCTAGC- *NheI*, CTCGAG-*XhoI* ,TCTAGA *–XbaI .*
