# Supplementary material for: Modulation of Catalytic Activity in Multi-Domain Protein Tyrosine Phosphatases
Source: PLoS One. 2011 Sep 13;6(9):e24766. doi: 10.1371/journal.pone.0024766 (PMC3172300; doi:10.1371/journal.pone.0024766)
Supplement: Table S2 — Kinetic parameters obtained for the dephosphorylation of various substrates by the active constructs of DLAR and PTP99A. (DOC) [file pone.0024766.s007.doc]

**Table S2 : Kinetic parameters obtained for the dephosphorylation of various substrates by the active constructs of DLAR and PTP99A .**

|  | **V_max_ ( μmole/min/mg )** | **K_m_ (μM )** | **K_cat_ ( sec^-1^ )** | **K_cat_/K_m_ ( sec^-1^M^-1^ )** |
| --- | --- | --- | --- | --- |
| **DLAR D1 D2** | | | | |
| **para-Nitro Phenyl Phosphate (pNPP)** | 6.19 ± 0.14 | 2.13 ± 0.15 x 10^3^ | 6.88 ± 0.16 | 3.23 ± 0.09 x 10^3^ |
| **Insulin Receptor TRDI(pY)ETDYYRK** | 24.31 ± 0.64 | 109.88 ± 7.82 | 28.99 ± 0.77 | 26.38 ± 0.04 x 10^4^ |
| **Cuticle TAEPD(pY)GALYE** | 26.48 ± 1.14 | 105.28 ± 12.38 | 31.58 ± 1.36 | 30.00 ± 0.07 x 10^4^ |
| **Myospheroid CDDS(pY)FGNKC** | 13.86 ± 0.39 | 115.77 ± 8.69 | 16.53 ± 0.47 | 14.28 ± 0.05 x 10^4^ |
| **Nervous Fingers VIGD(pY)VCRLCK** | 12.41 ± 0.68 | 75.49 ± 12.5 | 14.79 ± 0.81 | 19.59 ± 0.11 x 10^4^ |
| **Abelson RDDT(pY)TAHAG** | 3.39 ± 0.19 | 22.55 ± 4.88 | 4.05 ± 0.22 | 17.96 ± 0.16 x 10^4^ |
| **DLAR D1 D2HSS** | | | | |
| **para-Nitro Phenyl Phosphate (pNPP)** | 5.84 ± 0.15 | 3.30 ± 0.24 x 10^3^ | 6.49 ± 0.17 | 1.97 ± 0.10 x 10^3^ |
| **Insulin Receptor TRDI(pY)ETDYYRK** | 4.01 ± 0.1 | 45.27± 4.1 | 4.77 ± 0.13 | 10.54 ± 0.06 x 10^4^ |
| **Cuticle TAEPD(pY)GALYE** | 10.61 ± 0.38 | 161.55 ± 13.8 | 12.65 ± 0.46 | 7.83 ± 0.05 x 10^4^ |
| **Myospheroid CDDS(pY)FGNKC** | 3.11 ± 0.12 | 63.92 ± 7.99 | 3.7 ± 0.15 | 5.79 ± 0.08 x 10^4^ |
| **Nervous Fingers VIGD(pY)VCRLCK** | 7.9 ± 0.35 | 134.23 ± 14.9 | 9.42 ± 0.42 | 7.02 ± 0.07 x 10^4^ |
| **Abelson RDDT(pY)TAHAG** | 0.6 ± 0.01 | 12.38 ±1.07 | 0.72 ± 0.01 | 5.82 ± 0.07 x 10^4^ |
| **DLAR D1** | | | | |
| **para-Nitro Phenyl Phosphate (pNPP)** | 20.56 ± 0.34 | 1.00 ± 0.08 x 10^3^ | 16.10 ± 0.27 | 16.03 ± 0.11 x 10^3^ |
| **Insulin Receptor TRDI(pY)ETDYYRK** | 117.76 ± 3.28 | 59.47 ± 5.6 | 89.39 ± 2.62 | 150.31 ± 0.06 x 10^4^ |
| **Cuticle TAEPD(pY)GALYE** | 77.6 ± 1.96 | 56.54 ± 4.64 | 61.98 ± 1.56 | 109.62 ± 0.06 x 10^4^ |
| **Myospheroid CDDS(pY)FGNKC** | 29.58 ± 0.84 | 78.51 ± 6.67 | 23.66 ± 0.67 | 30.14 ± 0.06 x 10^4^ |
| **Nervous Fingers VIGD(pY)VCRLCK** | 37.5 ± 1.4 | 38.21± 5.05 | 30 ± 1.12 | 78.51 ± 0.09 x 10^4^ |
| **Abelson RDDT(pY)TAHAG** | 4.95 ± 0.59 | 18.19 ± 8.98 | 3.96 ± 0.68 | 21.77 ± 0.32 x 10^4^ |
| **PTP99A D1 D2** | | | | |
| **para-Nitro Phenyl Phosphate (pNPP)** | 24.89 ± 0.52 | 2.27 ± 0.17 x 10^3^ | 33.80 ± 0.59 | 14.92 ± 0.98 x 10^3^ |
| **Insulin Receptor TRDI(pY)ETDYYRK** | 45.98 ± 0.83 | 35.75 ± 2.08 | 56.86 ± 1.03 | 163.84 ± 0.74 x 10^4^ |
| **Cuticle TAEPD(pY)GALYE** | 17.10 ± 0.66 | 112.42 ± 10.14 | 21.15 ± 0.82 | 18.81 ± 1.29 x 10^4^ |
| **Myospheroid CDDS(pY)FGNKC** | 1.09 ± 0.03 | 3.06 ± 0.36 | 1.35 ± 0.03 | 44.1 ± 1.22 x 10^4^ |
| **Nervous Fingers VIGD(pY)VCRLCK** | 8.89 ± 0.38 | 179.86 ± 15.02 | 11.01 ± 0.46 | 6.12 ± 0.12 x 10^4^ |
| **Abelson RDDT(pY)TAHAG** | 2.59 ± 0.07 | 16.29 ± 1.55 | 3.21 ± 0.08 | 19.97 ± 0.15 x 10^4^ |
| **PTP99A D1** | | | | |
| **para-Nitro Phenyl Phosphate (pNPP)** | 0.22 ± 0.01 | 5.60 ± 0.23 x 10^3^ | 0.14 ± 0.01 | 0.20 ± 0.05 x 10^2^ |
| **Insulin Receptor TRDI(pY)ETDYYRK** | 2.37 ± 0.16 | 1017 ± 161.84 | 1.58 ± 0.11 | 15.53 ± 0.22 x 10^2^ |
| **Cuticle TAEPD(pY)GALYE** | 1.26 ± 0.04 | 309.04 ± 33.32 | 0.84 ± 0.04 | 27.18 ± 0.15 x 10^2^ |
| **Myospheroid CDDS(pY)FGNKC** | 2.72 ± 0.13 | 887.25 ± 102.23 | 1.81 ± 0.08 | 20.40 ± 0.59 x 10^2^ |
| **Nervous Fingers VIGD(pY)VCRLCK** | 4.42 ± 0.14 | 1361.68 ± 94.68 | 2.94 ± 0.10 | 21.61 ± 0.24 x 10^2^ |
| **Abelson RDDT(pY)TAHAG** | 0.83 ± 0.05 | 121.69 ± 26.61 | 0.55 ± 0.04 | 45.19 ± 0.29 x 10^2^ |
