# Supplementary material for: Modulation of Catalytic Activity in Multi-Domain Protein Tyrosine Phosphatases
Source: PLoS One. 2011 Sep 13;6(9):e24766. doi: 10.1371/journal.pone.0024766 (PMC3172300; doi:10.1371/journal.pone.0024766)
Supplement: Table S3 — Sequence motifs defining the PTP domain of DLAR and PTP99A. (DOC) [file pone.0024766.s008.doc]

**Table S3 :** **Sequence motifs defining the PTP domain of DLAR and PTP99A.**

|  | **Motifs consensus** | **DLAR D1** | **DLAR D2** | **PTP99A D1** | **PTP99A D2** | **PTP1B** |
| --- | --- | --- | --- | --- | --- | --- |
| **Motif 1** | Nxx(K/R)NRY | NKSKNRY | NKHKNRL | NKRKNRY | VNSIKNR | NKNRNRY |
| **Motif 2** | DxxR(V/I)xL | DHSRVQL | ESSRVYL | DHSRVHL | EGSRVHL | DHSRIKL |
| **Motif 3** | DYINA(N/S) | DYINAN | DYVNAS | DYINAN | DYINAS | DYINAS |
| **Motif 4** | (F/Y)(I/V)IAxQGP | YVATQGP | YIAAQGP | FIGTQGP | FIVTQHP | ILTQGP |
| **Motif 5** | TxxDFWx(M/L/V)xW(E/Q) | TFVDFNRMCWE | AAEDFWRMLWE | TFDCFWRMIWE | TIKDFWQMVWD | TCGHFWEMVWE |
| **Motif 6** | (I/L/V)(V/I)MxT | IVMMT | VVMLT | IVMIT | VVLLS | VVMLN |
| **Motif 7** | KCxxYWP | KCDQYWP | KCFQYWP | KCDMYWT | -QF-WPD | KCAQYWP |
| **Motif 8** | (Y/F)xxWPDxGxP | FTAWPDHGVP | FIDWPEGGVP | YTNWPDHGTP | CPSWPEMSNP | YTTWPDFGVP |
| **Motif 9** | Pxx(V/I)HCSAGxGR(T/S)G | PVIVHCSAGVGRTG | PITVHCSAGVGRSG | PIVVHCSAGVGRTG | PIVIVDRIGGAQA | PVVVHCSAGIGRSG |
| **Motif 10** | (V/I/L)QTxxQYxF | VQTEDQYIF | VQTEDQYHF | VQTEEQYIF | WTSSEDIRV | IQTADQLRF |

Sequence motifs defining the PTP domain of DLAR and PTP99A. Listed alongside are the corresponding residues in PTP1B.
