# Supplementary material for: Modulation of Catalytic Activity in Multi-Domain Protein Tyrosine Phosphatases
Source: PLoS One. 2011 Sep 13;6(9):e24766. doi: 10.1371/journal.pone.0024766 (PMC3172300; doi:10.1371/journal.pone.0024766)
Supplement: Table S4 — RMSF for the ten sequence motifs defining the PTP domains of DLAR and PTP99A. (DOC) [file pone.0024766.s009.doc]

**Table S4 :** **RMSF for the ten sequence motifs defining the PTP domains of DLAR and PTP99A.**

|  | **DLAR D1** | | **DLAR D2** | | **PTP99A D1** | | **PTP99A D2** | |
| --- | --- | --- | --- | --- | --- | --- | --- | --- |
|  | **C_alpha** | **All_element** | **C_alpha** | **All_element** | **C_alpha** | **All_element** | **C_alpha** | **All_element** |
| **Motif 1** | **0.260** | **0.597** | **0.292** | **0.751** | **0.214** | **0.718** | **0.377** | **0.980** |
| **Motif 2** | **0.250** | **0.714** | **0.225** | **0.540** | **0.268** | **0.709** | **0.267** | **0.632** |
| **Motif 3** | **0.172** | **0.404** | **0.199** | **0.468** | **0.175** | **0.475** | **0.270** | **0.619** |
| **Motif 4** | **0.184** | **0.481** | **0.215** | **0.472** | **0.195** | **0.418** | **0.192** | **0.489** |
| **Motif 5** | **0.214** | **0.485** | **0.288** | **0.596** | **0.199** | **0.492** | **0.213** | **0.600** |
| **Motif 6** | **0.166** | **0.579** | **0.170** | **0.606** | **0.209** | **0.592** | **0.211** | **0.685** |
| **Motif 7** | **0.220** | **0.463** | **0.233** | **0.524** | **0.232** | **0.529** | **0.171** | **0.584** |
| **Motif 8** | **0.320** | **0.604** | **0.423** | **0.795** | **0.361** | **0.627** | **0.577** | **1.014** |
| **Motif 9** | **0.258** | **0.504** | **0.285** | **0.527** | **0.284** | **0.540** | **0.376** | **0.727** |
| **Motif 10** | **0.194** | **0.566** | **0.192** | **0.532** | **0.231** | **0.602** | **0.252** | **0.741** |

.

RMSF values were calculated over the entire simulation time and are listed separately for the C-α backbone and the all-atom perturbations.
