# Supplementary material for: Modulation of Catalytic Activity in Multi-Domain Protein Tyrosine Phosphatases
Source: PLoS One. 2011 Sep 13;6(9):e24766. doi: 10.1371/journal.pone.0024766 (PMC3172300; doi:10.1371/journal.pone.0024766)
Supplement: Table S5 — Residue numbers for the functionally important residues (FIR) as they occur in the sequence of DLAR and PTP99A, and as they are seen in the homology models of the RPTPs used in the Molecular Dynamics Simulations. (DOC) [file pone.0024766.s010.doc]

**Table S5:** **Residue numbers for the functionally important residues (FIR) as they occur in the sequence of DLAR and PTP99A, and as they are seen in the homology models of the RPTPs used in the Molecular Dynamics Simulations**.

|  | **DLAR** | | **PTP99A** | |
| --- | --- | --- | --- | --- |
| **Role of the Residues** | **Residue number in the actual Protein sequence** | **Residues number in the homology model used for MD simulations** | **Residue number in the actual Protein sequence** | **Residues number in the homology model used for MD simulations** |
|  | D1 Domain | | D1 Domain | |
| Phospho-peptide Binding residues | Tyr 1503 | Tyr 55 | Tyr 509 | Tyr 59 |
|  | Ala 1504 | Ala 56 | Leu 510 | Leu 60 |
|  | Asn 1505 | Asn 57 | Asn 511 | Asn 61 |
|  | Val 1506 | Val 58 | Ile 512 | Ile 62 |
|  | Glu 1576 | Glu 128 | Glu 583 | Glu 133 |
| WPD loop | Trp 1636 | Trp 188 | Trp 649 | Trp 199 |
|  | Asp 1638 | Asp 190 | Asp 651 | Asp 201 |
| Active site residues | His 1639 | His 191 | His 652 | His 202 |
|  | His 1669 | His 221 | His 682 | His 232 |
|  | Cys 1670 | Cys 222 | Cys 683 | Cys 233 |
|  | Ser 1671 | Ser 223 | Ser 684 | Ser 234 |
|  | Ala 1672 | Ala 224 | Ala 685 | Ala 235 |
|  | Val 1674 | Val 226 | Val 687 | Val 237 |
|  | Arg 1676 | Arg 228 | Arg 689 | Arg 239 |
|  | Thr 1677 | Thr 229 | Thr 690 | Thr 240 |
| Arginine loop | Arg 1706 | Arg 258 | Arg 719 | Arg 269 |
|  | Arg 1709 | Arg 261 | Arg 722 | Arg 272 |
| Glutamine loop | Gln 1714 | Gln 266 | Gln 727 | Gln 277 |
|  | Gln 1718 | Gln 270 | Gln 731 | Gln 281 |
|  | D2 domain | | D2 domain | |
| Phospho-peptide Binding residues | Leu 1792 | Leu 344 | Arg 795 | Arg 345 |
|  | Val 1793 | Val 345 | Gly 796 | Gly 346 |
|  | His 1794 | His 346 | Ala 797 | Ala 347 |
|  | Ile 1795 | Ile 347 | Ile 798 | Ile 348 |
|  | Glu 1865 | Glu 417 | Asp 868 | Asp 418 |
| WPD loop | Trp 1925 | Trp 477 | Trp 926 | Trp 476 |
|  | Glu 1927 | Glu 479 | Glu 928 | Glu 478 |
| Active site residues | Gln 1928 | Gln 480 | Met 929 | Met 479 |
|  | His 1960 | His 512 | Val 957 | Val 507 |
|  | Cys 1961 | Cys 513 | Asp 958 | Asp 508 |
|  | Ser 1962 | Ser 514 | Arg 959 | Arg 509 |
|  | Ala 1963 | Ala 515 | Tyr 960 | Tyr 510 |
|  | Val 1965 | Val 517 | Gly 962 | Gly 512 |
|  | Arg 1967 | Arg 519 | Gln 964 | Gln 514 |
|  | Ser 1968 | Ser 520 | Ala 965 | Ala 515 |
| Arginine loop | Arg 1997 | Arg 549 | His 994 | His 544 |
|  | Arg 2000 | Arg 552 | Arg 997 | Arg 547 |
| Glutamine loop | Gln 2005 | Gln 557 | Thr 1002 | Thr 552 |
|  | Gln 2009 | Gln 561 | Asp 1006 | Asp 556 |
